# Supplementary material for: Solid-Phase Synthesis of an Insect Pyrokinin Analog Incorporating an Imidazoline Ring as Isosteric Replacement of a trans Peptide Bond
Source: Molecules. 2021 May 28;26(11):3271. doi: 10.3390/molecules26113271 (PMC8198379; doi:10.3390/molecules26113271)
Supplement: Supplementary file 1 [file molecules-26-03271-s001.zip › molecules-1206145-supplementary.pdf]

Solid Phase Synthesis of an Insect Pyrokinin Analog Incorporating an Imidazoline Ring as Isosteric Replacement of a *trans* Peptide Bond

Krzysztof Kaczmarek [1,2,*], Barbara Pacholczyk-Sienicka [2], Łukasz Albrecht [2], Janusz Zabrocki [1,2] and Ronald J. Nachman [1,*]

1 Insect Control and Cotton Disease Research Unit, ARS, U.S. Department of Agriculture, 2881 F-B Road, College Station, TX 77845 USA
2 Institute of Organic Chemistry, Lodz University of Technology, 90-924 Łódź, Poland; barbara.pacholczyk @p.lodz.pl (B.P.-S.); lukasz.albrecht@p.lodz.pl (Ł.A.); janusz.zabrocki@p.lodz.pl (J.Z.)
* Correspondence: krzysztof.kaczmarek@p.lodz.pl (K.K.); ron.nachman@usda.gov (R.J.N.); Tel.: +48 42 6313156 (K.K.); Tel.: +001-979-324-4805 (R.J.N.)

SUPPLEMENTARY MATERIAL

Materials and Methods

Purification and Amino Acid Analysis

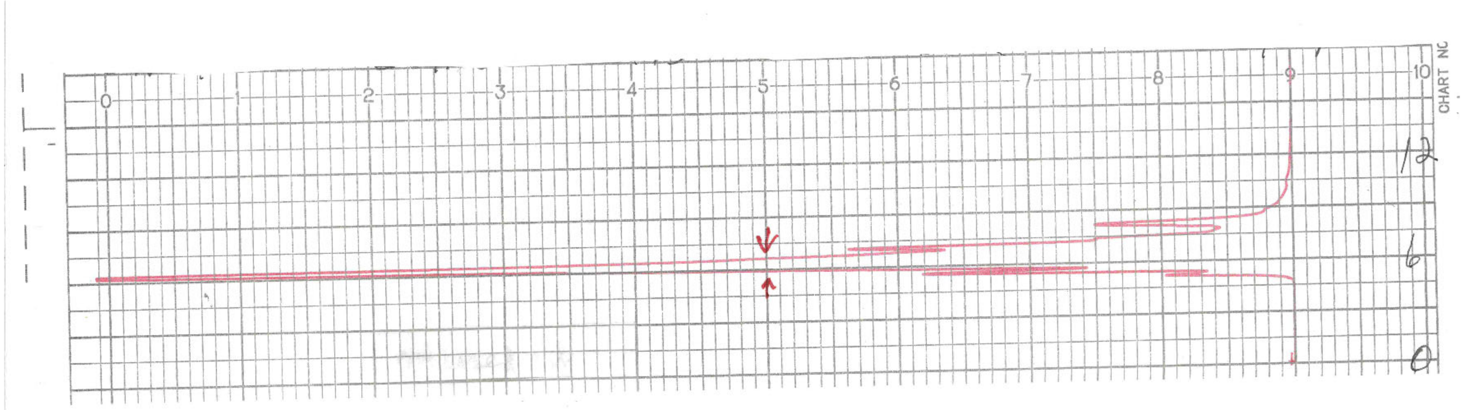

Figure S1. HPLC trace of partially purified product PPK-Jo (from reversed phase C_{18}) injected on Waters Protein Pack column for further separation.

HRMS measurements

Elemental Composition Report

Single Mass Analysis

Tolerance = 5.0 PPM / DBE: min = -50.0, max = 80.0
Element prediction: Off
Number of isotope peaks used for i-FIT = 5

Monoisotopic Mass, Even Electron Ions
200 formula(e) evaluated with 2 results within limits (up to 50 closest results for each mass)
Elements Used:
C: 0-40 H: 0-60 N: 0-14 O: 0-10
181128_peptydA 14 (0.160) Cm (13:17-(3:8+32:72))
TOF MS ES+

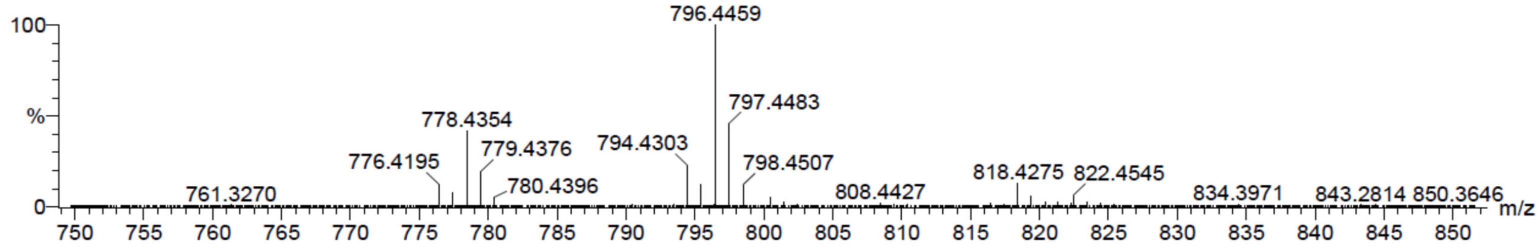

Minimum:				-50.0				
Maximum:		15.0	5.0	80.0				
Mass	Calc. Mass	mDa	PPM	DBE	i-FIT	Norm	Conf(%)	Formula
778.4354	778.4364	-1.0	-1.3	16.5	913.9	1.490	22.54	C38 H56 N11 O7
	778.4324	3.0	3.9	12.5	912.7	0.255	77.46	C33 H56 N13 O9

Figure S2. HRMS of PPK-Jo displayed with computed possible empirical formulas.

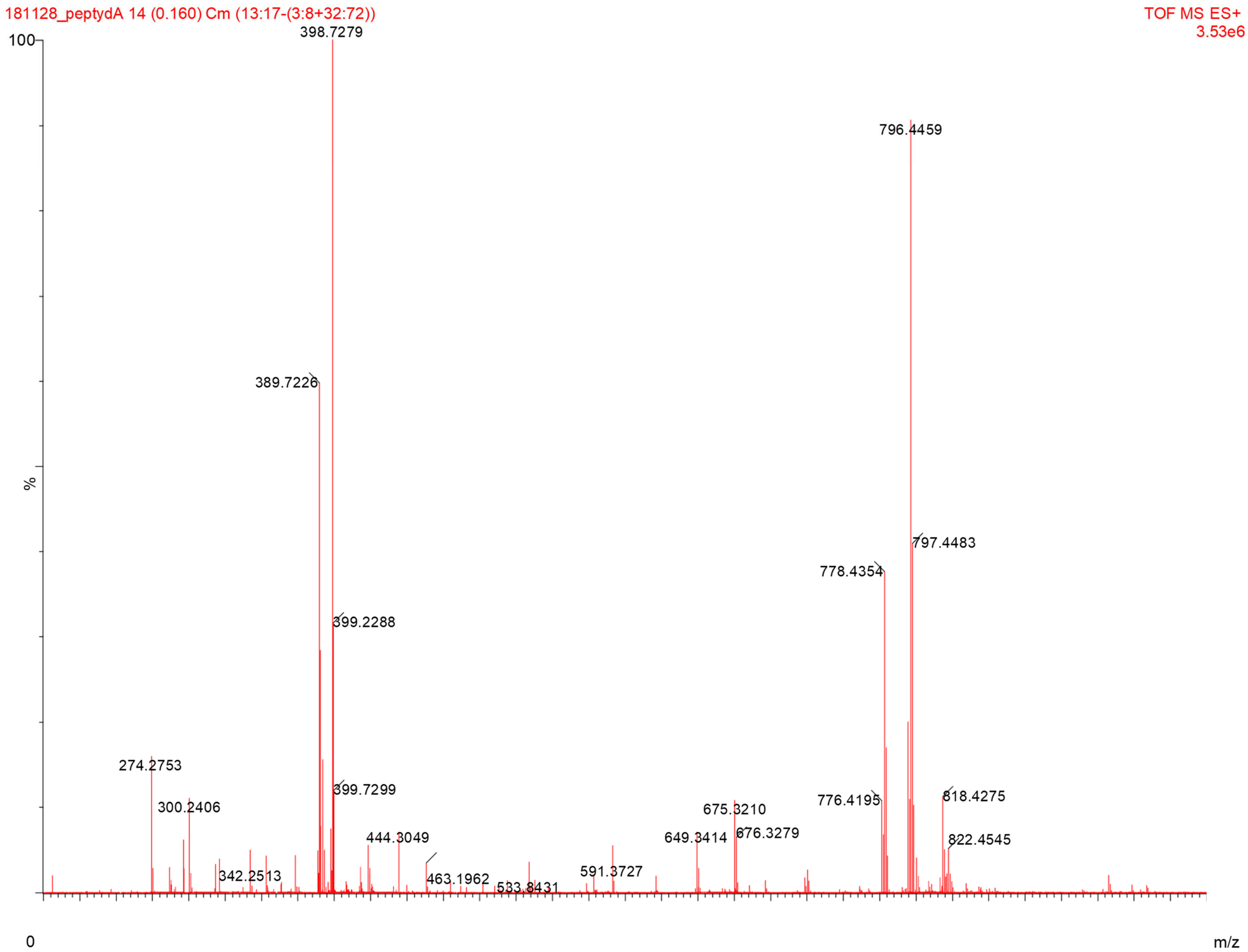

Figure S3. HRMS measurements. Calc. for $C_{38}H_{56}N_{11}O_7$ = 778.4264 [MH^+], found 778.4254 [MH^+] and 379.7226 [MH_2^{2+}] for single and doubly charged ions, respectively.

NMR experiments

	H/C	δ, ppm $[H \rightarrow N]$	δ, ppm $[H \rightarrow {}^{\alpha}C]$	δ, ppm $[H \rightarrow {}^{\beta}C]$	δ, ppm $[H \rightarrow {}^{\gamma}C]$	δ, ppm $[H \rightarrow {}^{\delta}C]$	δ, ppm $[H \rightarrow {}^{\varepsilon}C]$	δ, ppm $[C = O]$	δ, ppm $[NH_2]$	δ, ppm $[CH_3]$	δ, ppm $[OH]$
Arg	H	8.03	4.34	1.76, 1.54	1.45	3.10	-	-	3.42	-	-
	C	-	55.01	24.75	25.65	40.14	-	173.50	-	-	-
Leu	H	-	4.25	1.60, 1.47	1.27	0.85, 0.89	-	-	3.44	-	-
	C	-	51.28	41.39	25.19	22.09, 23.35	-	172.91	-	-	-
Phe	H	8.13	4.51	2.92, 2.83	7.16	7.28	7.23	-	-	-	-
	C	-	50.84	34.37	129.51	127.40	126.20	173.01	-	-	-
Tyr	H	8.08	4.23	3.03, 3.16	6.61	6.95	-		-	-	4.58
	C	-	51.43	41.94	115.31	130.13	-	170.82	-	-	-
Ac	H	-	-	-	-	-	-	-	-	1.93	-
	C	-	-	-	-	-	-	169.60	-	21.63	-

Imidazoline	δ, ppm $[NH]$	δ, ppm $[CH_{ring}]$	δ, ppm $[CH_{2ring}]$	δ, ppm $[C =_{ring}]$	δ, ppm $[CH]$	δ, ppm $[CH_3]$
H	7.48	4.82	3.72, 4.10	-	4.08	1.44
C		56.40	51.92	146.50	47.30	25.20

Table S1. ^{1}H and ^{13}C chemical shifts for all atoms in PPK-Jo.

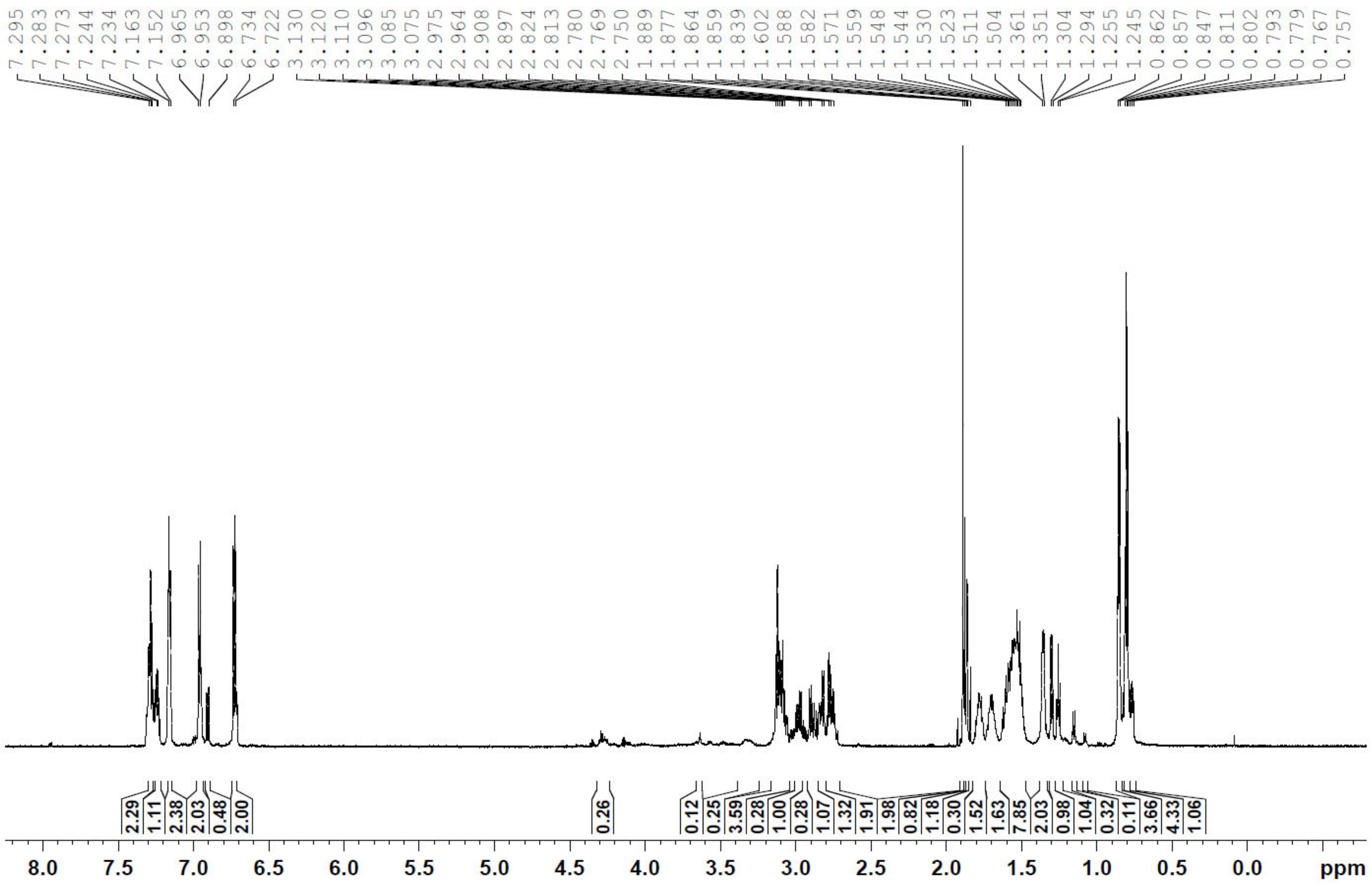

Figure S4. ^1H NMR spectrum of PPK-Jo in D_2O.

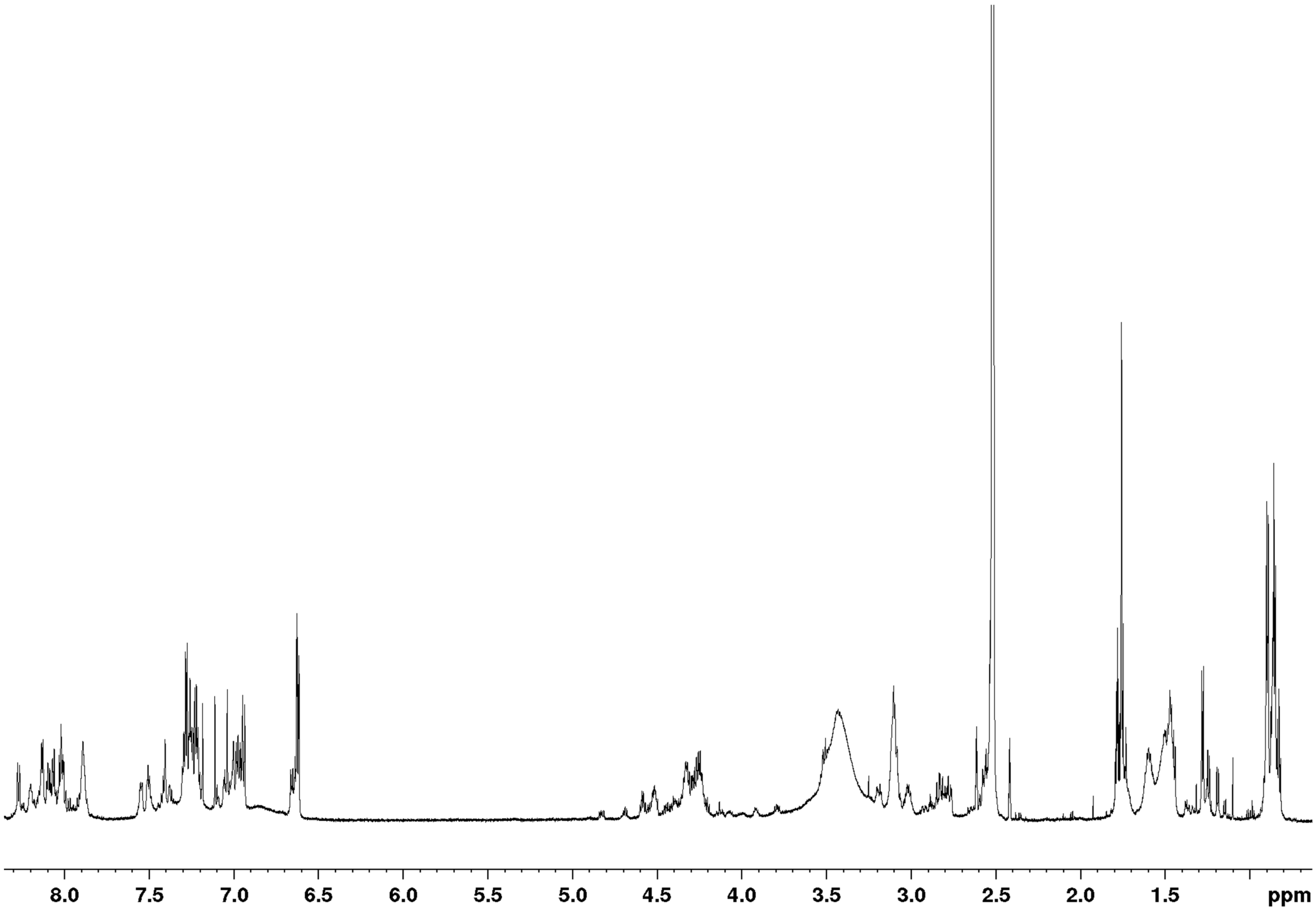

Figure S5. ^{1}H NMR spectrum of PPK-Jo in DMSO.

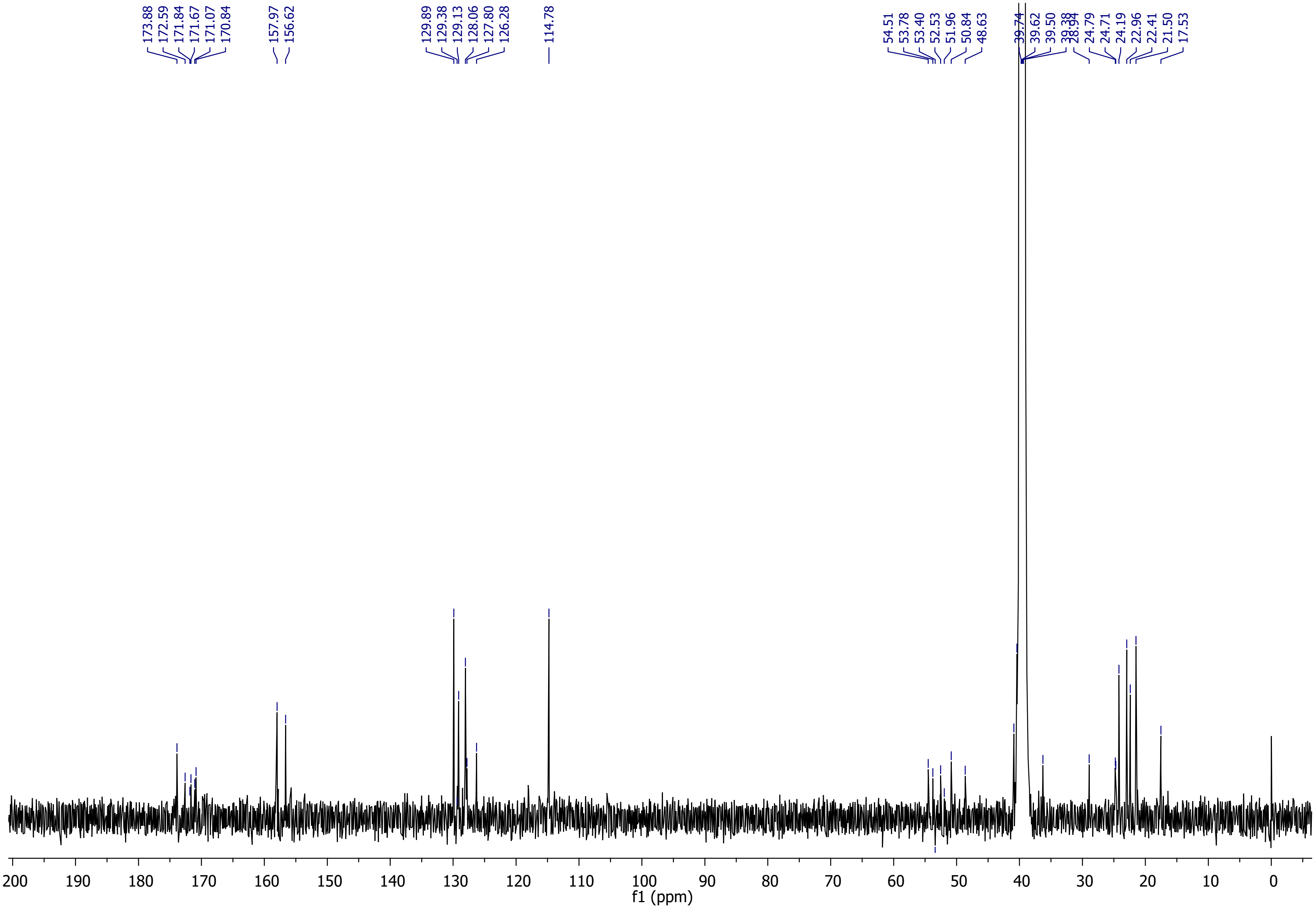

Figure S6. ^{13}C NMR spectrum of PPK-Jo in DMSO.

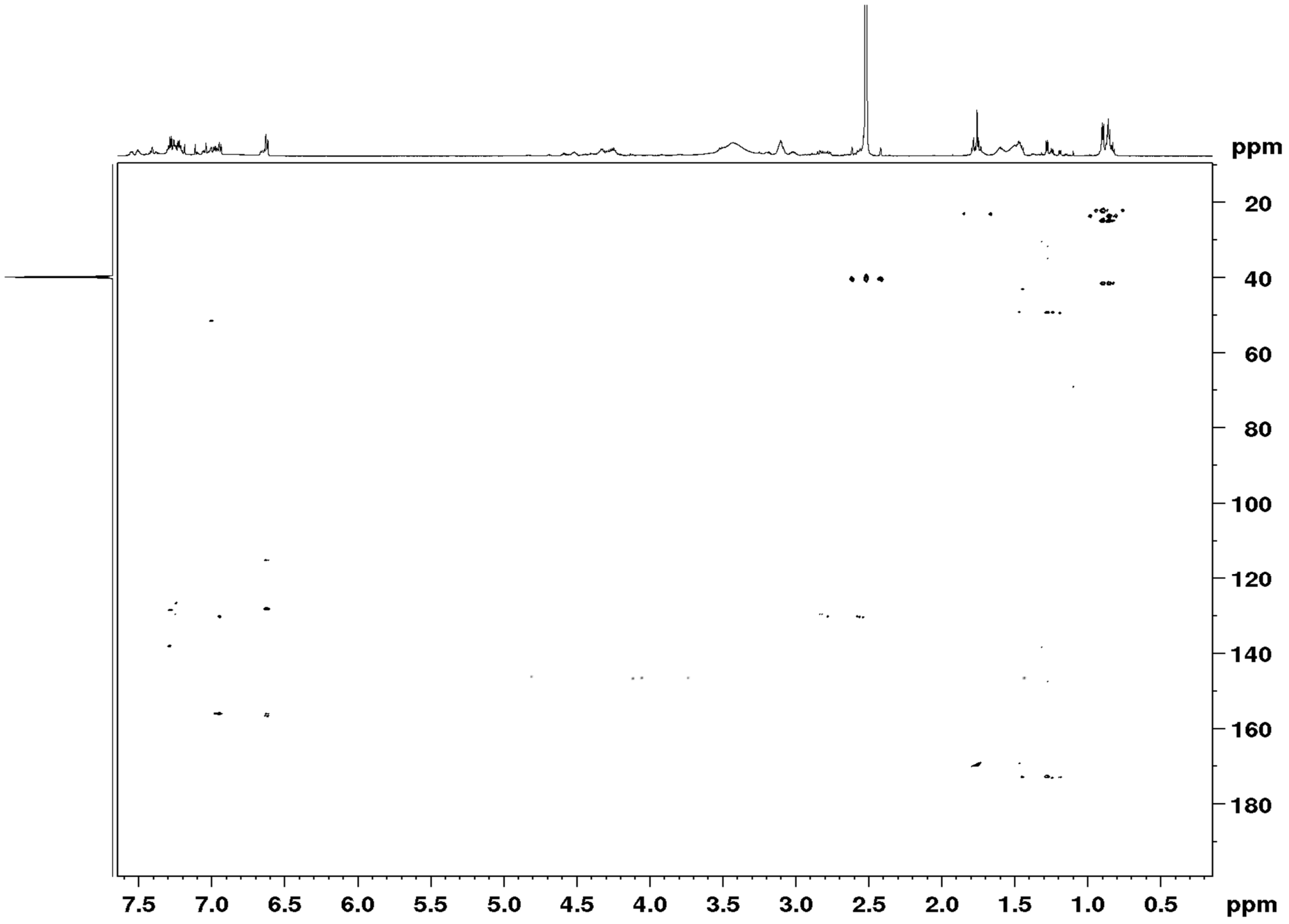

Figure S7. ^{1}H-^{13}C HMBC spectrum of PPK-Jo in DMSO.

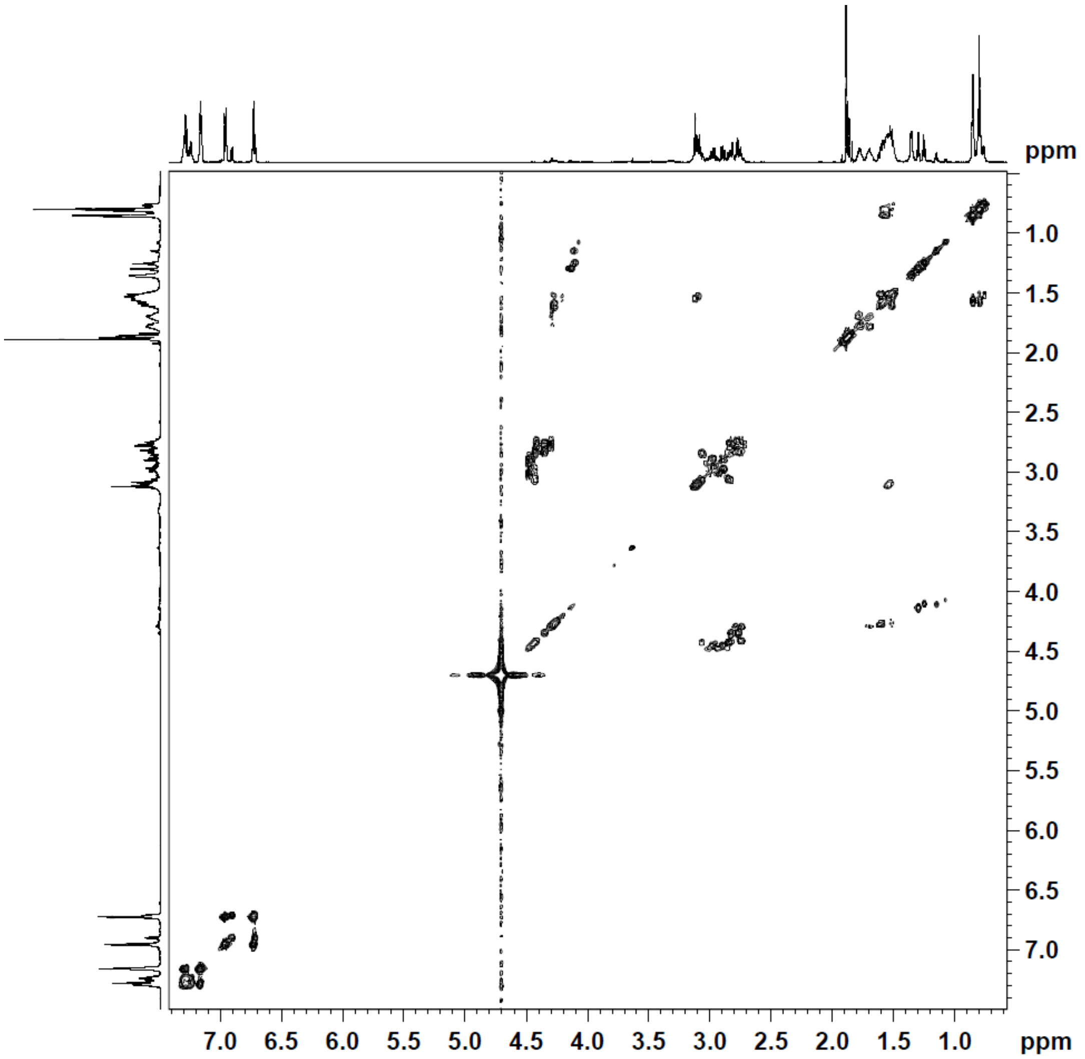

Figure S8. ^1H-^1H COSY spectrum of PPK-Jo in D_2O.

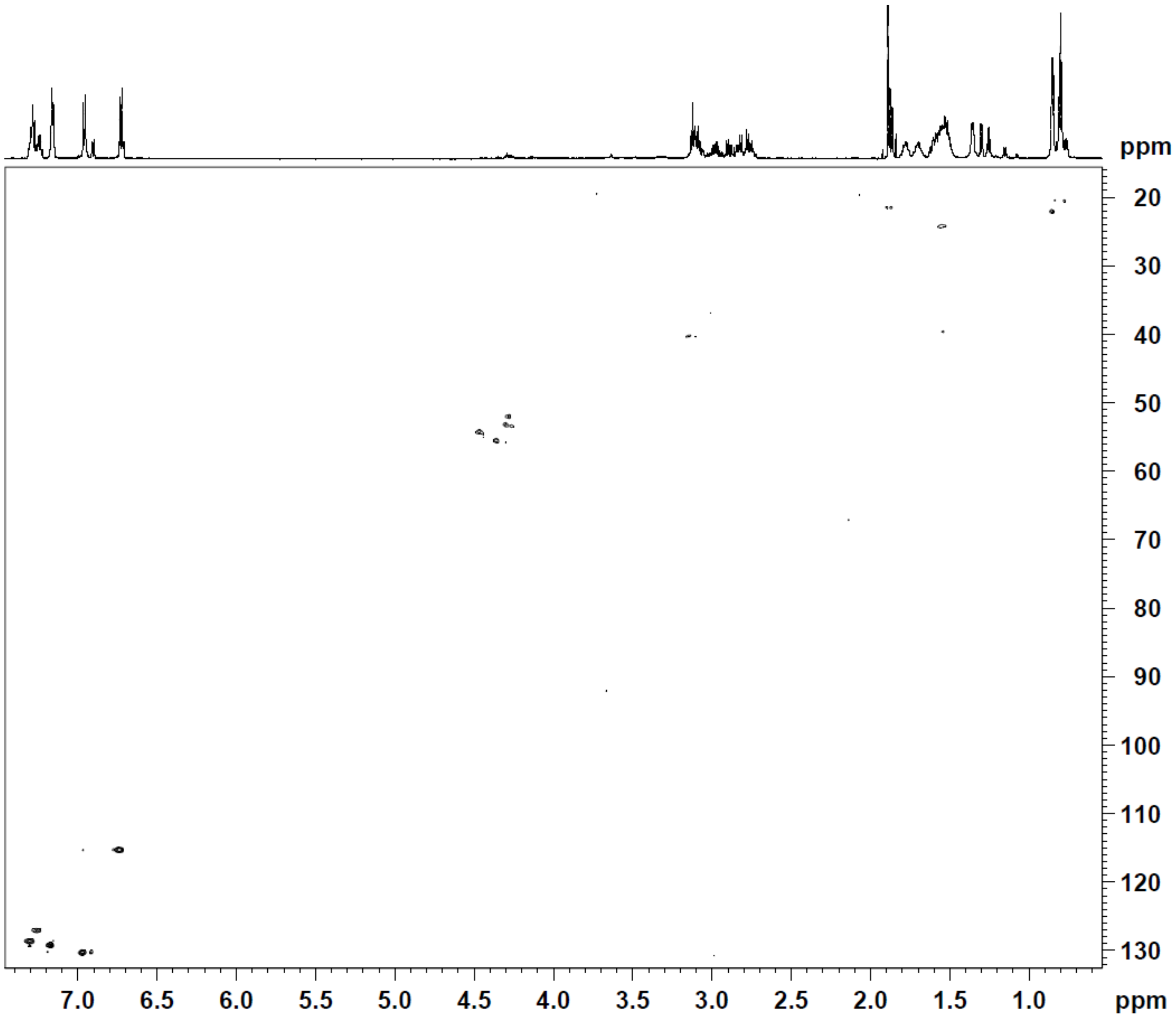

Figure S9. ^{1}H-^{13}C HSQC spectrum of PPK-Jo in D_2O.

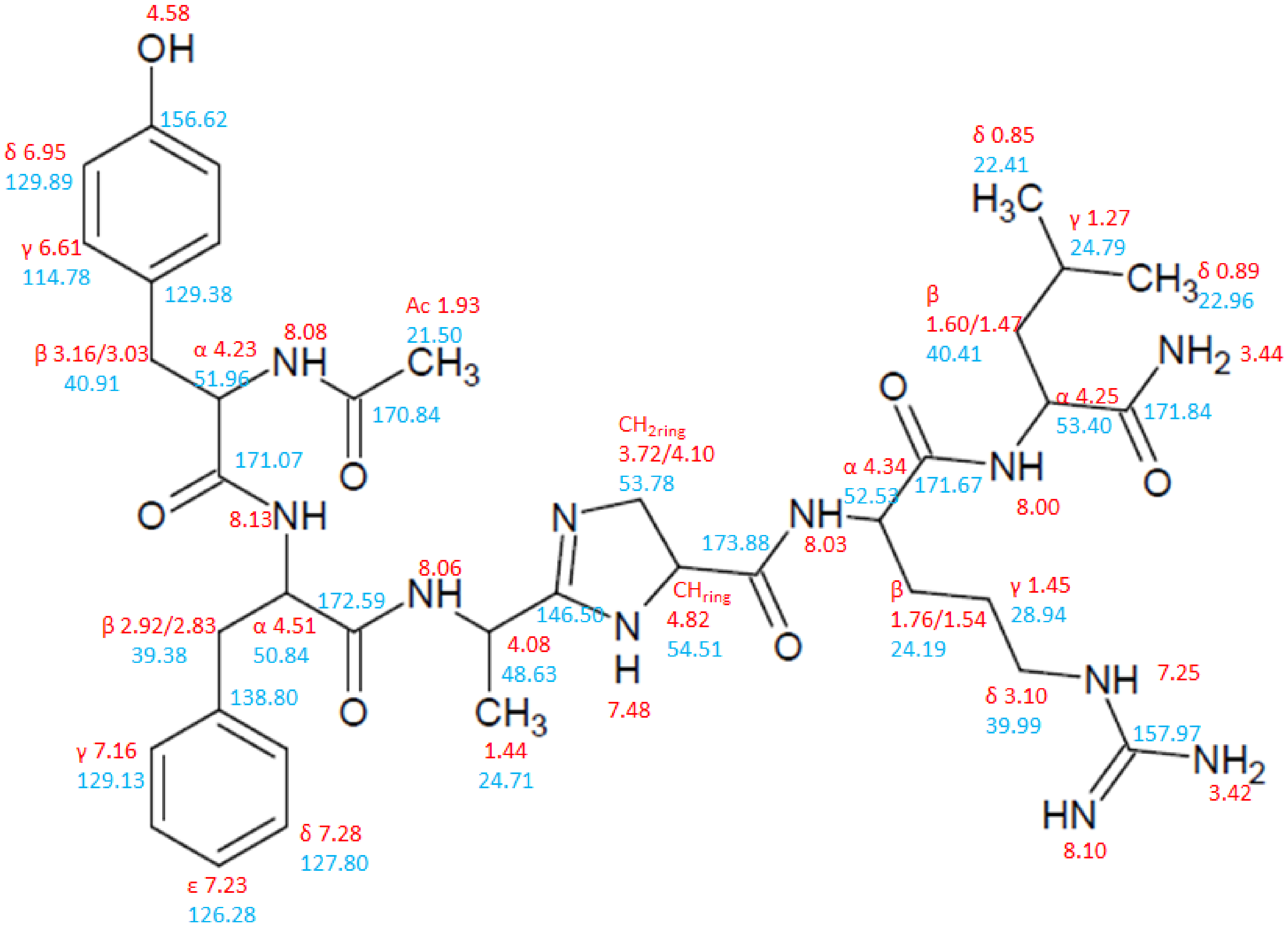

Figure S10. Structure of PPK-Jo with shifts of H and C atoms displayed.
